# Growth, biochemical response and liver health of juvenile barramundi (*Lates calcarifer*) fed fermented and non-fermented tuna hydrolysate as fishmeal protein replacement ingredients

**DOI:** 10.7717/peerj.4870

**Published:** 2018-06-05

**Authors:** Muhammad A.B. Siddik, Janet Howieson, Ilham Ilham, Ravi Fotedar

**Affiliations:** 1 School of Molecular and Life Sciences, Curtin University, Bentley, Australia; 2 Department of Fisheries Biology and Genetics, Patuakhali Science and Technology University, Patuakhali, Bangladesh; 3 Department of Aquaculture, Jakarta Fisheries University, Jakarta, Indonesia

**Keywords:** Barramundi, GPx activity, Fermentation, Tuna hydrolysate, Biochemical response

## Abstract

Conventional aquaculture feed materials available in Australia are expensive, which has prompted the search for alternatives that would be cost-effective and locally available. The present study was undertaken in order to maximize the use of a tuna hydrolysate (TH), which was produced locally from the tuna-processing discards. The growth performance, biochemical status, antioxidant capacity and liver health of juvenile barramundi (*Lates calcarifer*) were assessed. Two series of isonitrogenous and isocaloric diets labelled as TH_50_, TH_75_ (non-fermented tuna hydrolysate) and FTH_50,_ FTH_75_ (fermented tuna hydrolysate) were formulated to replace FM at 50% and 75%, respectively. A basal diet without the TH supplementation was used as a control. The experimental diets were fed to the triplicate groups of fish three times a day for 56 days. The results of the experiment revealed that fish fed on both fermented and non-fermented TH-containing diets significantly reduced (*p* < 0.05) the final body weight, weight gain and specific growth rate compared to the control. The highest apparent digestibility coefficients for dry matter, protein and lipid were obtained in the control group, and decreased with the increasing level of TH in the diets. However, the whole-body proximate compositions and the blood biochemical indices of fish were not affected by the TH inclusion in the diets. The fish fed on TH diets of TH_50_, FTH_50_ and TH_75_ exhibited reduced (*p* < 0.05) glutathione peroxidase (GPx) activity compared to the control; whereas the FTH_75_ exhibited no difference with the control. The excessive inclusion of TH in the diets of TH_75_ and FTH_75_ resulted in cytoplasmic vacuolization, with an increased amount of lipid accumulation, and necrosis in the liver tissue. These results indicated that the replacement of the FM protein with TH at 50% and 75% inclusion levels negatively affected the growth performance, feed utilization, and digestibility in juvenile barramundi; and it also increased the potential risk of hepatic failure in the fish. Further investigation is, therefore, required in order to optimize the TH levels in the fish diets which would be suitable for the growth of fish, as well as for maintaining the enhanced biochemical response in juvenile barramundi.

## Introduction

Fish-processing industries produce a large volume of fish waste across the globe as the fillets are often the only desired product in the market (Knuckey et al., 2004). As a result of the environmental concerns and the increased cost of waste disposal, options for waste utilization are being considered in order to maximize the use of the discarded fish waste, which will generate a more economic return from the same harvest, and cause less impact on the environment. In recent years, fish protein hydrolysates (FPH) obtained from a variety of low-value fish by-products, such as skin, fins, frames, heads, viscera, trimmings, and roe, have received significant consideration in the aqua feeds for their variety of uses, for example, as protein replacements (Kim et al., 2014; Ospina-Salazar et al., 2016), supplements (Bui et al., 2014; Khosravi et al., 2015a), attractants (Ho et al., 2014), palatability enhancers (Suresh, vasagam & Nates, 2011), and immunostimulants (Bui et al., 2014). FPH, because of their short-chain peptides and well-balanced amino acids, are easily absorbed by animals and facilitate the biological nutrients uptake (Carvalho et al., 2004). Although beneficial effects, such as growth enhancement, feed utilization, and survival, have been reported for the inclusion of FPH at moderate levels (5% to 30%) in the fish diets, these bioactive substances have also been reported to result in an increase in the innate immune response (Liang et al., 2006), gut enzymatic activity (Cahu et al., 1999), disease resistance (Khosravi et al., 2015b), and stimulation of digestibility (Kousoulaki et al., 2013). In addition to good nutritive values and functional properties, some authors have reported that FPH plays a substantial role in the fish health as promoters for antioxidant and antimicrobial activities (Bougatef et al., 2010; Chalamaiah et al., 2012).

As an FPH, tuna hydrolysate (TH) is a nutrient-rich dietary supplement, which has been used in the aquafeeds since a long time (Tacon, Hasan & Subasinghe, 2006). The availability of TH in Australia initiated this study, as several studies over the years have demonstrated that the TH could serve as an ideal protein source in the aqua diets for replacing FM, or it may be used as a feed palatability enhancer resulting in a higher feed intake, or for the stimulation of the immune and digestive systems of the fish (Kolkovsksie, Czesny & Dabrowski, 2000). The optimum level of TH inclusion in the aqua diets is species-specific and most of the studies have reported inclusion levels of up to 30% (Kim et al., 2014; Ospina-Salazar et al., 2016). It has been reported previously that higher inclusion levels may lead to imbalanced absorption of the amino acids and thus, saturate the peptide transportation system in the fish (Carvalho et al., 2004). It is, therefore, of interest to search for the techniques which could possibly improve the nutritional values of TH, potentially allowing the incorporation of higher amounts of TH in the diets, in order to enhance the bioactive, antioxidant, and antimicrobial properties, on a species-specific level.

In order to improve the nutritional value of the feed ingredients, several techniques such as fermentation, advanced bioprocessing, soaking, and germination may be applied (Vo et al., 2015). Among these techniques, fermentation is the widely used one, for improving the nutritional quality of the feed ingredients, as well as for enhancing the palatability of the feed ingredients. Fermentation has also been associated with the reduction of ash content in the feed ingredients which may stabilize the pellet inside water (Fagbenro, Kim Jauncey & Haylor, 1994), improvement in the digestibility of various feed ingredients (Samaddar, Kaviraj & Saha, 2015), and the removal of growth limiting factors in both plant and animal feed ingredients (Shamna et al., 2017). It has also been reported that fermentation by yeast and lactic acid bacteria induced immune modulation which may enhance the growth, non-specific immune response, and disease resistance in the host fish (Giri, Sukumaran & Oviya, 2013).

Barramundi (*Lates calcarifer*) is widely distributed across the coastal areas of the Asia-Pacific region, all the way up to Papua New Guinea and northern Australia (Glencross, 2006; Siddik et al., 2016). This species has excellent attributes for use in aquaculture, including rapid growth rate, high consumer preference, a competitive market price, an accepted taste, and the ability to be cultured in a wide range of environments including ponds, sea cages, and recirculating aquaculture systems (Schipp, Bosmans & Humphrey, 2007). The expansion of barramundi aquaculture has resulted in an increased pressure on the environment and on the sustainable supply of high-quality, less-expensive FM. This, in turn, has led a demand for further research in the area of finding alternatives to FM protein. The by-products of fish processing could serve as suitable alternatives to FM, because these are less expensive and locally available, in addition to being environmentally safe. According to the research conducted to date, a maximum of 50% FM has been successfully replaced with tuna muscle by-product hydrolysate in juvenile Japanese flounder (*Paralichthys olivaceus*), without suppressing the growth performance and feed utilization (Uyan et al., 2006). However, no study has so far evaluated the suitability of fermented TH at a higher inclusion level, such as 50% and above, as an alternative to fishmeal protein. We have been refining the fermentation technique for TH for several years now and our preliminary data with the other host species have provided us with a certain direction for increasing the inclusion levels of non-fermented and fermented TH. Therefore, the present study was conducted to assess the efficacy of fermented as well as non-fermented TH as a protein source, in terms of their effects on the growth performance, antioxidant capacity, biochemical status, and liver health in juvenile barramundi.

## Materials and Methods

### Ethics statement

The growth trial of the fish was performed in accordance with the Australian code of conduct for the use and care of animals for scientific purposes. The procedures and protocols used in this experiment for treating fish were approved by the Animal Ethics Committee of Curtin University, Australia (Approval Number: AEC_2015_41).

### Fish and rearing conditions

Juvenile barramundi, obtained from the Australian Centre for Applied Aquaculture Research, Fremantle, WA, Australia were used for the experiment. Prior to the commencement of feeding, the fish were acclimated to the laboratory conditions for one week; during that period they were fed on a commercial barramundi diet containing 470 g protein kg^−1^ diet and 20.0 MJ kg^−1^ dietary gross energy three times a day. The barramundi juveniles, with a mean initial weight of 6.75 ± 0.16 g fish^−1^, were stocked randomly into fifteen tanks (capacity: 250 L each), at a stocking density of 20 fish tank^−1^. The water in all the tanks used for this experiment was recirculated independently from an external bio-filter with a water refreshment capacity of 10 L min^−1^ and was constantly aerated to ensure sufficient levels of dissolved oxygen.

The water temperature, salinity, pH, and the level of dissolved oxygen in the water were monitored daily, and the levels of nitrite and ammonia were monitored twice a week. The water quality parameters maintained during the experimental period were as follows: water temperature = 27.8–29.7 °C, salinity = 31–38 ppt, pH = 7.20–8.10, dissolved oxygen concentration = 5.65–7.45 mg L^−1^, nitrite <0.25 mg L^−1^, and total ammonia nitrogen <0.5 mg L^−1^. The room for the experiment was maintained under a 12-h light–12-h dark cycle, using *automatic indoor-light* switches (Clipsal, Australia). Prior to feeding, the daily feed ration for the fish was divided into three equal portions, and then the fish were hand-fed to satiation with the respective experimental diets, three times a day, at 0,800 h, 1,300 h, and 1,800 h. After 0.5 h of each time, the uneaten feed was removed from the tank by siphoning, transferred to aluminum cups, and dried to constant weight, in order to evaluate the feed conversion ratio.

### Preparation of fermented TH

Tuna hydrolysate (TH) supplied by SAMPI, Port Lincoln, Australia, was fermented by following the technique proposed by Himawan et al. (2016). Baker’s yeast *Saccharomyces cerevisiae* (Instant dried yeast, Lowan^®^, Glendenning, Australia), and *Lactobacillus casei* in the form of a skim-milk product (Yakult^®^, Tokyo, Japan), were used at 10% and 5% (cell density for *Lactobacillus casei* = 3 × 10^6^ CFU g^−1^ meal) of the total weight of the meal mixture, respectively, and distilled water was used at approximately 70% of the total weight of the meal ingredients; all these ingredients were homogenized together in a food mixer. The mixture was transferred into an Erlenmeyer flask, which was then covered with aluminum foil and incubated at 30 °C for 4 d. Following this, the fermented product was dried in an oven at 60 °C for 24 h, and then used as a feed ingredient.

### Experimental diets

All the feed ingredients used in this study, except TH, were obtained from Specialty Feeds Pty. Ltd, Great Eastern Highway, Western Australia; TH was provided by SAMPI, Port Lincoln, Australia. The diets were formulated to fulfill the nutritional requirements of the juvenile barramundi set by the NRC (2011). Two series of experimental diets, isonitrogenous and isocaloric, were formulated for the juvenile barramundi, containing approximate 47% crude protein (CP) and 20 MJ kg^−1^ gross energy (GE), respectively. The diets were labeled as TH_50_ and TH_75_ (non-fermented TH), and FTH_50_ and FTH_75_ (fermented TH). The diets were formulated to replace FM at 50% and 75% inclusion levels of TH/FTH. A basal diet with FM as the sole protein source was used as the control diet. Another set of diets was formulated with the addition of 5 g chromic oxide (Cr_2_O_3_) per kg of diet as an inert marker for assessing the digestibility in the fish (Cr_2_O_3_; Thermo Fisher Scientific, Scoresby, VIC, Australia). All the test diets were processed with the addition of water to about 35% mash dry weight of the mixed ingredients in order to form a dough. The dough was passed through a mincer in order to create pellets of desired diameter (3 mm). The moist pellets were oven dried at 60 °C for 48 h and then, cooled at room temperature, sealed in plastic bags, and stored at −15 °C until further use. The formulations and the proximate compositions of the experimental diets are presented in Table 1.

**Table 1:** Ingredients and proximate composition of the experimental diets.

	Control	TH_50_	TH_75_	FTH_50_	FTH_75_
Ingredients (g kg^−1^)^a^					
Fish meal	610.00	305.00	152.50	305.00	152.50
Tuna hydrolysate	–	415.00	589.50	454.00	606.50
Wheat flour	266.00	152.00	110.00	113.00	75.00
Wheat starch	20.00	20.00	20.00	20.00	20.00
Fish Oil	30.00	30.00	30.00	30.00	30.00
Limestone (CaCO_3_)	2.00	2.00	2.00	2.00	2.00
Salt (NaCL)	2.00	2.00	2.00	2.00	2.00
Vitamin Premix^b^	1.00	1.00	1.00	1.00	1.00
Casein	63.00	70.00	90.00	70.00	108.00
Cellulose	6.00	3.00	3.00	3.00	3.00
Proximate composition (% dry matter basis)					
Crude protein	47.42	47.31	47.20	47.08	47.29
Crude lipid	10.00	10.15	10.67	10.19	10.09
Ash	13.04	8.48	6.15	8.61	6.26
GE (MJ kg^−1^)	19.98	20.10	20.19	20.24	20.26

**Notes.**

^a^ Supplied by Specialty Feeds, Perth, Australia. ^b^ Contains the following (as g kg^−1^ of premix): iron, 10; copper, 1.5; iodine, 0.15; manganese, 9.5; zinc, 25; vitamin A retinol, 100 IU; vitamin D3, 100 IU; vitamin E, 6.25; vitamin K, 1.6; vitamin B1, 1; vitamin B2, 2.5; niacin, 20; vitamin B6, 1.5; calcium, 5.5; biotin, 0.1; folic acid, 0.4; inositol, 60; vitamin B12, 0.002; choline, 150; and ethoxyquin, 0.125.

TH tuna hydrolysate FTH fermented tuna hydrolysate GE gross energy MJ kg^−1^ mega joule per kilogram

### Digestibility assessment

In order to estimate the apparent digestibility coefficients (ADCs) for dry matter, protein, and lipid, fecal matter was collected using the stripping technique (Austreng, 1978), 15 h post feeding, prior to terminating the feeding trial. All the fecal matters collected from each tank within each period were pooled and frozen at −20 °C immediately. Prior to commencing the analysis, the fecal samples were oven-dried to a constant weight at 105 °C. The chromium oxide content in the diet formulations and the fecal samples was analyzed by the method described by Cho, Slinger & Bayley (1982). The ADCs for each nutritional component in the test diets were calculated based on the formula given below:

\begin{eqnarray*}& & \text{ADC}=100-100\ast [\text{marker in feces}(\text{%})/\text{marker in diet}(\text{%})]^{\ast } \end{eqnarray*}

\begin{eqnarray*}& & \quad [\text{nutrient in feces}(\text{%})/\text{nutrient in diet}(\text{%})] \end{eqnarray*}

### Sampling and chemical analysis

At the termination of the trial, the fish fasted for 24 h. The fish were then anesthetized with 5 mg L^−1^ of AQUI-S (Australia), followed by bulk weighing in order to assess the final body weight (FBW), specific growth rate (SGR), feed conversion ratio (FCR), and survival rate for the fish. Blood samples were collected from the caudal veins of three fish from each tank, using a 1-mL plastic syringe and a 22G × 1∕2″ straight needle. The extracted blood sample was transferred to heparinized tubes for the analysis of biochemical indices of blood, such as hemoglobin (Hb) content, hematocrit concentration, leucocrit concentration, and the glutathione peroxidase (GPx) enzyme activity. Hematocrit (Hct,%) and leucocrit (%) concentrations were determined by using the standard McLeay and Gordon’s method (McLeay & Gordon, 1977), following a centrifugation at 2,000 rpm for 5 min. The hemoglobin (Hb,%) content was determined by using an Hb kit (Randox Laboratories, Antrim, United Kingdom). The GPx activity (GPx units g^−1^ Hb) in the red blood cells was assessed by using the Ransel RS–505 assay kit (Randox, Antrim, United Kingdom).

The moisture, crude protein, crude lipid, ash, and gross energy contents in the experimental diets were assessed using the methods and procedures given by the Association of Official Analytical Chemists (AOAC, 2006). Moisture in the samples was determined by oven-drying to a constant weight at 105 °C; ash content was determined by combustion at 550 °C for 24 h in an electric furnace (Carbolite, Sheffield, UK); crude protein content (N × 6.25) was determined by following the Kjeldahl method, using a Kjeltec Auto 1030 analyzer (Foss Tecator, Höganäs, Sweden); crude lipid was analyzed by following the Soxhlet technique, using a Soxtec System HT6 (Tecator, Höganäs, Sweden); and the gross energy content was determined by using a bomb calorimeter (Heitersheim, Germany). The composition of amino acids, except tryptophan, in the tested diets, was analyzed by using high-performance liquid chromatography (HPLC), following an acid hydrolysis.

### Histopathology

In order to analyze the histopathological conditions, one liver segment from an individual fish, i.e., six liver segments from each treatment were sampled. The liver tissue samples were excised and preserved in 10% buffered formalin until they were processed using the standard histological procedures. The blocks of the designated samples were dehydrated in 100% ethanol, and then, embedded in paraffin wax. The sections of approximately 5 µm in size were cut and stained with Hematoxylin-Eosin (H&E) stain, for histological examination under a light microscope (BX40F4, Olympus, Tokyo, Japan). All the samples were prepared using the standard histological techniques (Luna, 1968).

### Calculations

The fish fasted for 24 h prior to weighing and sampling, and the following parameters were measured after the growth trial of 56 d:

\begin{eqnarray*}\text{Weight gain, WG}=[\text{(mean final body weight}\text{--- mean initial body weight)}] \end{eqnarray*}

\begin{eqnarray*} /[\text{mean initial body weight}]. \end{eqnarray*}

\begin{eqnarray*}\text{Specific growth rate, SGR}~(\text{%}/\text{day})=[\text{(ln mean final body weight}\text{--- in mean}\nonumber\\\displaystyle  \text{initial body weight)/number of days}]\times 100 \end{eqnarray*}

\begin{eqnarray*}\text{Feed intake, FI}=\text{dry feed consumed/number of fish.} \end{eqnarray*}

\begin{eqnarray*}\text{Feed conversion ratio, FCR}=\text{dry feed fed/wet weight gain.} \end{eqnarray*}

\begin{eqnarray*}\text{Condition factor, CF}(\mathrm{g}~({\mathrm{cm}}^{3})^{-1})=[(\text{body weight, g})/(\text{length, cm})^{3}]\times 100. \end{eqnarray*}

\begin{eqnarray*}\text{Hepatosomatic index, HSI (%)}=\text{[(liver weight, g)/(body weight, g)]}\times 100. \end{eqnarray*}

\begin{eqnarray*}\text{Viscerosomatic index, VSI (%)}=\text{[(visceral weight, g)/(body weight, g)]}\times 100. \end{eqnarray*}

\begin{eqnarray*}\text{Survival rate, SR}=\text{(final number of fish/initial number of fish)}\times 100 \end{eqnarray*}

\begin{eqnarray*}\text{Skewness}=[1/n{\Sigma }_{i=1}^{n}({x}_{i}-\overline{x})^{3}]/[1/n{\Sigma }_{i=1}^{n}({x}_{i}-\overline{x})^{2}]^{3/2} \end{eqnarray*}

where Σ is the summation for all the observations (*x*_*i*_) within a sample, and $\overline{x}$ is the sample mean.

### Statistical analysis

The statistical analyses were performed using SPSS version 24 for Windows, IBM, Curtin University, Australia. The experimental data for growth performance, digestibility, hematological parameters, and body composition were subjected to one-way ANOVA, followed by Duncan’s multiple-range test. The data on length and weight distribution represented through skewness were executed together with the normal distribution test. Data values were expressed as a mean ± standard error in the triplicate tanks, and the threshold of statistical significance was set at *p* < 0.05.

## Results

### Growth performance, feed utilization and somatic indices

**Figure 1:**
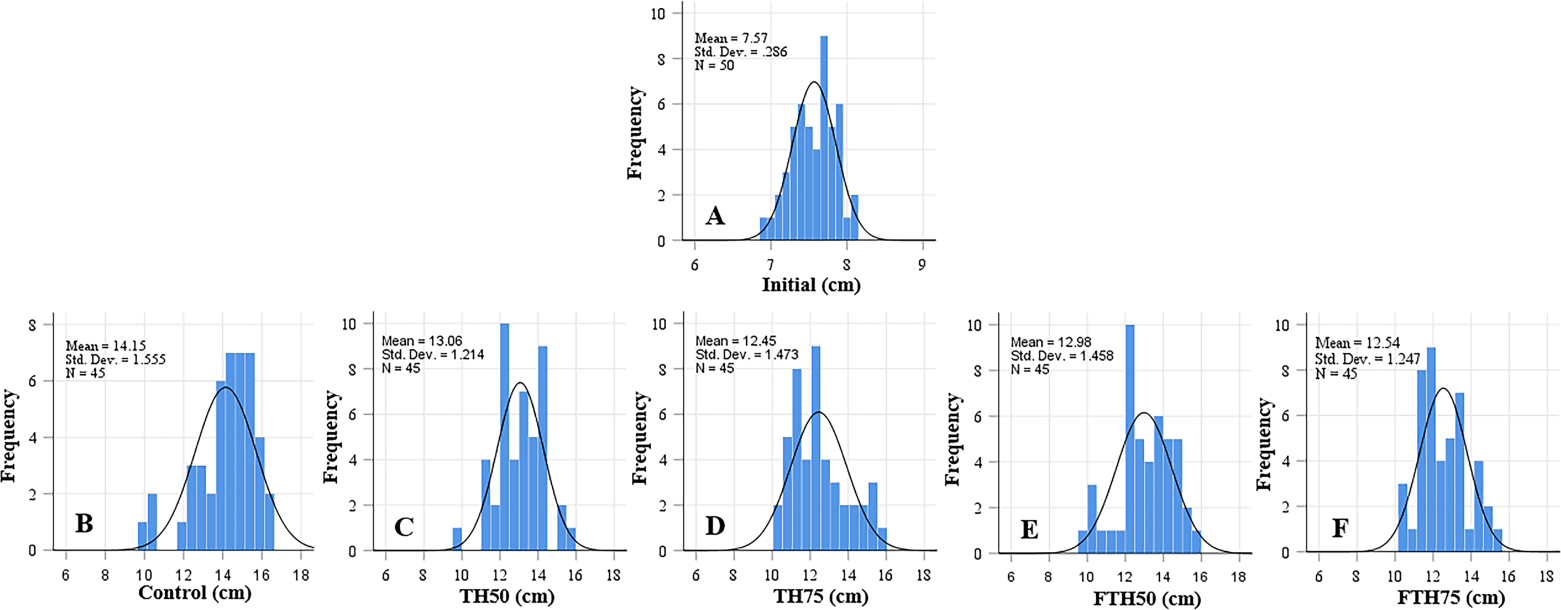
Length frequency distribution of initial fish (A) and fish fed on TH diets (B–F) without or with fermentation after 56 days. Frequency histograms of fish from different groups where control, TH_50_ and FTH_50_ fish showing negatively skewed curve indicate a higher proportion of large-body species, and the remaining groups of TH_75_ and FTH_75_ skewed positively indicate small-body species within the normal distribution. *n* = 50 for initial fish (A), *n* = 45 for each experimental treatment (B–F).

**Figure 2:**
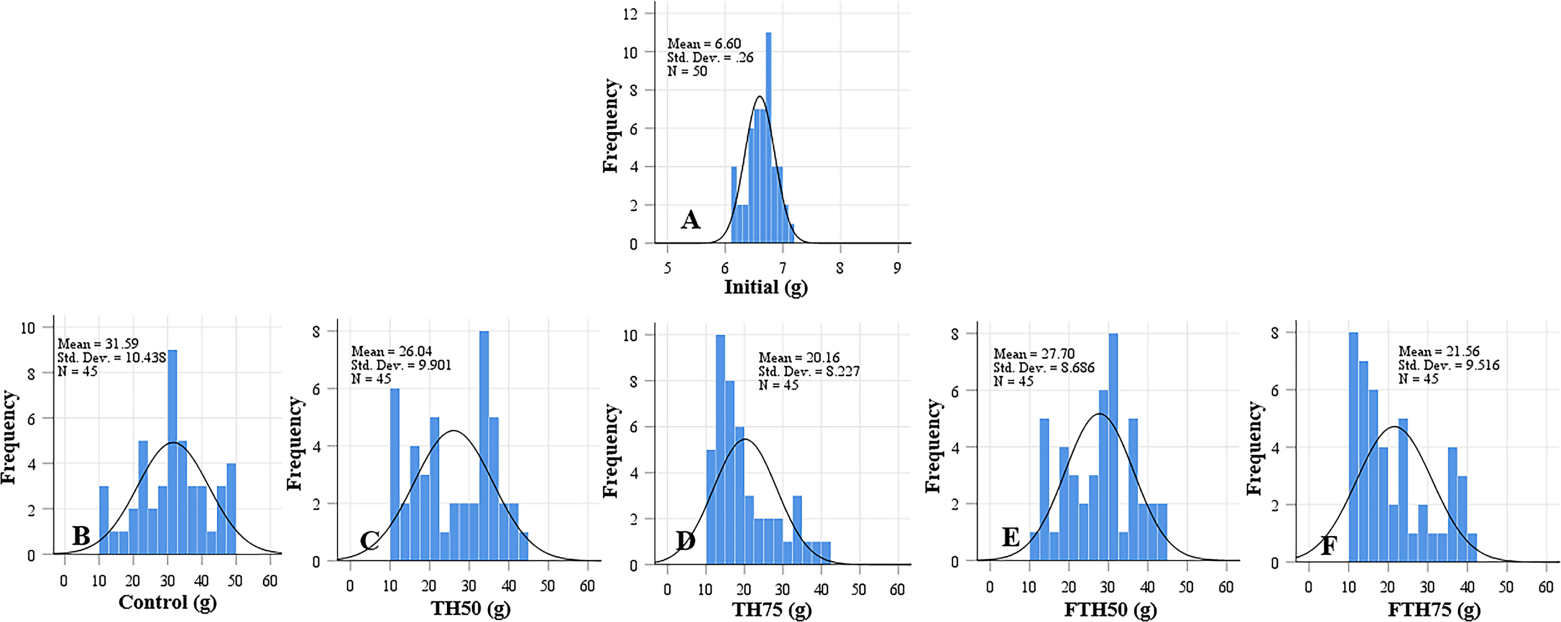
Weight distribution of initial fish and fish (A) and fish fed on TH diets (B–F) without or with fermentation after 56 days. Frequency distributions of control and TH_50_ and FTH_50_ fish skewed negatively indicates a higher proportion of large-body species and fish from TH_75_ and FTH_75_ groups skewed positively indicate small-body species within the distribution. *n* = 50 for initial fish (A), *n* = 45 for each experimental treatment (B–F).

The growth performance, feed utilization, and somatic indices for the juvenile barramundi fed on diets containing different inclusion levels of fermented and non-fermented TH for 56 d are presented in Table 2. At the end of the growth trial, it was observed that the growth performance parameters, including FBW, WG, and SGR, decreased as the levels of inclusion of TH and FTH in the diets increased. The control treatment demonstrated best growth performance, and the lowest growth performance was observed for the 75% replacement level in both fermented and non-fermented treatments. Similarly, highest FI was observed in the control group of fish, which was fed on the FM-based diet. The FCR was significantly higher in the fish fed on TH_75_ diet compared to the fish which were fed on all the other diets, except for the fermented-TH group of the same replacement level—FTH_75_. No significant difference was observed in the FCR among the groups of fish fed on the control, TH_50_, and FTH_50_ diets. The body indices, such as VSI, HSI, and CF, and the survival rate of the fish were not affected by the inclusion of TH or FTH in the diets. The distributions of length and weight of the juvenile barramundi fed on different diets are presented in Figs. 1 and 2, and the respective values for skewness are presented in Table 2. The variations in the length and weight of the fish that were fed on different diets were not significant compared to control. Negative skewness, in terms of length, was obtained for the control, TH_50_, and FTH_50_ groups; whereas, negative skewness, in terms of weight, was observed for the control and TH_50_ groups.

**Table 2:** Growth performance, feed utilization and somatic indices of juvenile barramundi fed on TH diets without or with fermentation for 56 days.

	Experimental diets					*P*-value
	Control	TH_50_	TH_75_	FTH_50_	FTH_75_	
*Growth performance parameters*						
FBW (g)	31.53^a^ ± 0.59	26.36^b^ ± 1.00	19.05^c^ ± 0.59	27.69^b^ ± 1.14	21.25^c^ ± 1.67	<0.001
WG (g)	25.17^a^ ± 0.31	19.40^b^ ± 1.10	12.38^c^ ± 0.66	20.91^b^ ± 1.15	14.25^c^ ± 1.18	<0.001
SGR (% day^−1^)	2.87^a^ ± 0.12	2.38^b^ ± 0.16	1.78^c^ ± 0.02	2.51^b^ ± 0.01	2.07^c^ ± 0.01	<0.001
FI (g fish^−1^day^−1^)	1.09^a^ ± 0.59	0.98^bc^ ± 0.59	0.92^c^ ± 0.59	1.00^b^ ± 0.59	0.94^c^ ± 0.59	<0.001
FCR	2.46^c^ ± 0.02	2.69^c^ ± 0.10	4.36^a^ ± 0.14	2.97^bc^ ± 0.45	3.64^ab^ ± 0.25	<0.05
VSI	8.95 ± 0.34	10.05 ± 0.36	9.02 ± 1.13	9.92 ± 0.63	9.77 ± 0.79	0.142
HSI	1.49 ± 0.15	2.15 ± 0.26	1.97 ± 0.45	2.20 ± 0.33	2.49 ± 0.38	0.277
CF	1.22 ± 0.03	1.05 ± 0.03	1.02 ± 0.09	0.99 ± 0.13	1.01 ± 0.03	0.277
SR	98.33 ± 1.67	95.00 ± 2.89	93.33 ± 1.67	96.67 ± 1.67	93.33 ± 1.67	0.364
*Size distribution statistics*						
Skewness for length	−1.087	−0.210	0.694	−0.375	0.280	
Skewness for weight	−0.132	−0.048	1.010	−0.159	0.719	

**Notes.**

Different superscript letters (a, b, c) in the same row denote significant differences (*p* < 0.05, 0.001) determined by one-way ANOVA followed by Duncan’s post hoc multiple range test.

FBW final bodyweight WG weight gain SGR specific growth rate FI feed intake FCR feed conversion ratio HSI hepatosomatic index VSI viscerosomatic index SR survival

Values are the mean of three replicate tanks (*n* = 3) ±  standard error.

### Digestibility

The digestibility coefficients for dry matter (DM), protein, and lipid in the juvenile barramundi fed on the diets that contained TH and FTH at various inclusion levels are enlisted in Table 3. As seen in Table 3, the higher inclusion levels of TH and FTH in the diets resulted in a significant decrease (*p* < 0.05) in the apparent digestibility coefficients (ADCs) for DM, protein, and lipid, with the lowest and the highest values of the ADCs for DM, protein, and lipid obtained for the TH_75_ and the control group, respectively.

**Table 3:** Apparent digestibility coefficients (%) of dry matter, crude protein and crude lipid of juvenile barramundi fed on TH diets without or with fermentation for 56 days.

	Experimental diets					*P*-value
	Control	TH_50_	TH_75_	FTH_50_	FTH_75_	
Dry matter	89.4^a^ ± 0.32	86.07^b^ ± 0.54	82.47^c^ ± 0.52	87.73^b^ ± 0.45	84.50^c^ ± 0.27	<0.001
Crude protein	93.97^a^ ± 0.51	92.0^b^ ± 0.76	90.01^c^ ± 0.74	92.41^ab^ ± 0.49	91.0^bc^ ± 0.35	<0.05
Crude lipid	95.90^a^ ± 0.15	93.48^bc^ ± 0.37	92.80^c^ ± 0.47	94.29^bc^ ± 0.38	93.43^bc^ ± 0.47	<0.05

**Notes.**

Different superscript letters (a, b, c) in the same row denote significant differences (*p* < 0.05, 0.001) determined by one-way ANOVA followed by Duncan’s post hoc multiple range test.

TH tuna hydrolysate FTH fermented tuna hydrolysate

Values are the mean of three replicated tanks (*n* = 3) ± standard error.

### Whole fish body composition

The whole-body proximate compositions (moisture, protein, lipid, and ash) and the energy content for the fish belonging to the different treatments are presented in Table 4. As seen in Table 4, the whole-body proximate composition and the gross energy of the juvenile barramundi were neither influenced by the TH types (fermented or non-fermented) nor by the levels of replacement (*p* > 0.05).

**Table 4:** Whole body proximate composition of juvenile barramundi fed on TH diets without or with fermentation for 56 days.

	Experimental diets					*P*-value
	Control	TH_50_	TH_75_	FTH_50_	FTH_75_	
Moisture (%)	74.57 ± 2.34	75.40 ± 2.42	77.63 ± 1.74	76.53 ± 2.32	77.43 ± 1.74	0.819
Protein (% DM)	14.67 ± 0.77	14.62 ± 0.15	13.22 ± 0.27	14.31 ± 1.14	13.50 ± 0.40	0.974
Lipid (% DM)	4.08 ± 0.09	3.86 ± 0.21	3.50 ± 0.27	3.55 ± 0.26	3.56 ± 0.32	0.991
Ash (% DM)	3.89 ± 0.07	3.71 ± 0.22	3.69 ± 0.04	3.82 ± 0.05	3.87 ± 0.16	0.693
GE (MJ kg^−1^)	18.56 ± 0.55	19.34 ± 1.17	17.18 ± 0.59	19.66 ± 2.32	18.80 ± 1.15	0.720

**Notes.**

Values without superscript letters (a, b, c) in the same row are insignificant (*p* < 0.05) determined by one-way ANOVA followed by Duncan’s post hoc multiple range test.

TH tuna hydrolysate FTH fermented tuna hydrolysate DM dry matter GE gross energy MJ kg^−1^ mega joule per kilogram.

Values are the mean of three replicated tanks (*n* = 3) ± standard error.

### Biochemical status

**Figure 3:**
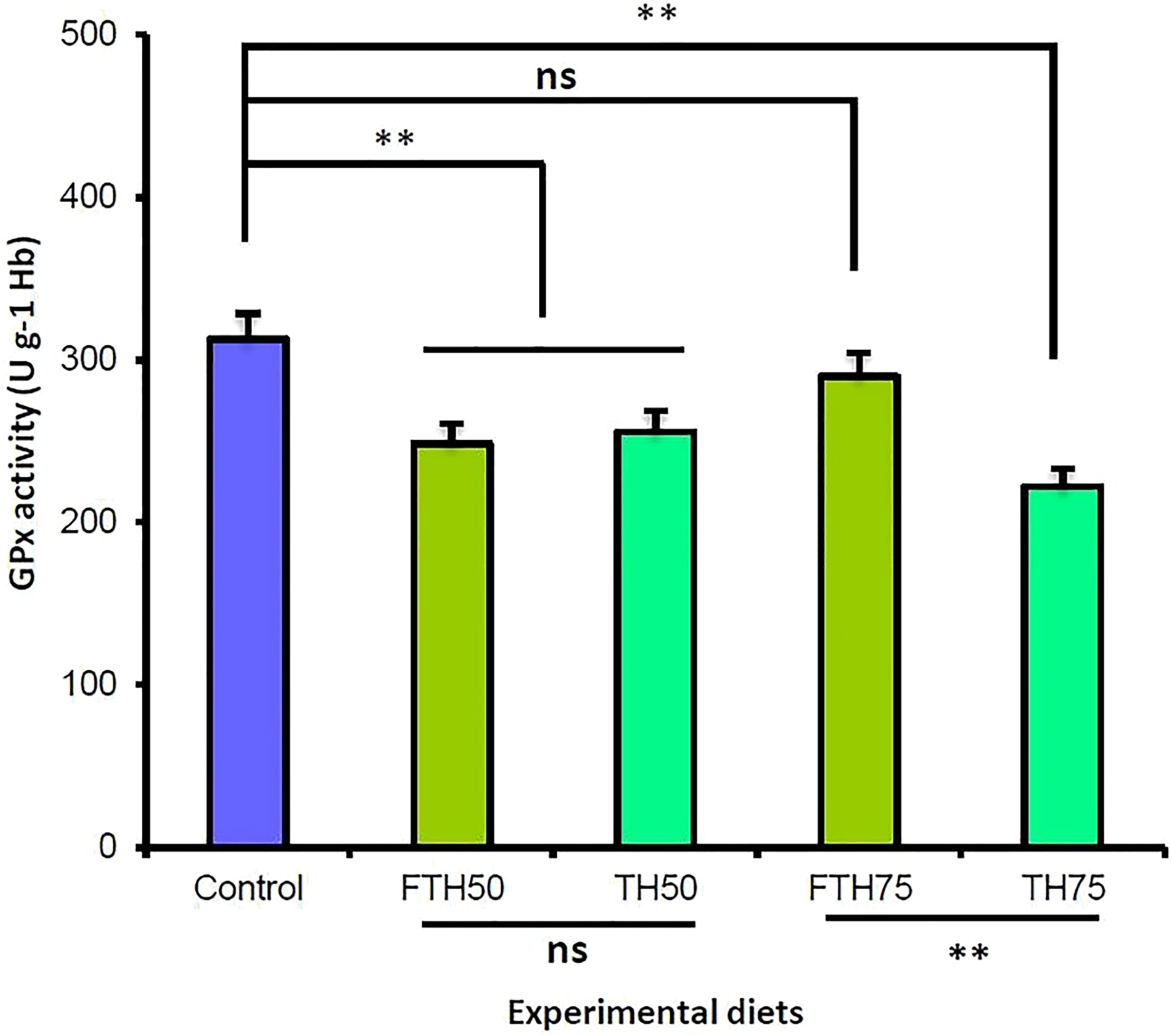
Glutathione peroxidase (GPx) activities of juvenile barramundi fed fermented and non-fermented tuna hydrolysate (TH) at various inclusion levels. Post-ANOVA Duncan’s multiple comparisons test was applied to compare GPx activities of fish fed on four experimental diets to the control. Values are the mean of three replicate tanks per treatment ± standard error. The significant difference was considered at *p* < 0.05. (**: significant; ns: non-significant).

The blood biochemical indices and the glutathione peroxidase (GPx) enzyme activity for the juvenile barramundi are presented in Table 5. The dietary inclusion of TH and FTH exhibited no significant effect on the blood hemoglobin, hematocrit, and leucocrit levels in the fish. However, the incremental inclusion of TH and FTH exhibited significant effects on the glutathione peroxidase (GPx) enzyme activity in the juvenile barramundi. The fish fed on the FM-replacement diets TH_50_, FTH_50_, and TH_75_ exhibited significantly reduced GPx activity compared to control; whereas, the 75% FM-replacement diet FTH_75_ exhibited no significant difference in the GPx activity compared to the control. Furthermore, the GPx activity was significantly increased in the fish fed on fermented diet compared to the fish fed on the non-fermented diet, when the replacement level was 75%; however, at 50% replacement level, the difference in the GPx activity was not significant between the fermented and the non-fermented diets (*p* > 0.05). The antioxidant glutathione peroxidase (GPx) activity values obtained for the juvenile barramundi belonging to different treatments are presented in Fig. 3.

**Table 5:** Blood biochemical parameters of juvenile barramundi fed on TH diets without or with fermentation for 56 days.

	Experimental diets					*P*-value
	Control	TH_50_	TH_75_	FTH_50_	FTH_75_	
Hb (g dl^−1^)	73.0 ± 6.25	67.0 ± 7.88	62.33 ± 1.86	63.67 ± 3.00	79.67 ± 6.69	0.241
Hct (%)	27.67 ± 2.19	24.0 ± 2.00	25.0 ± 0.88	28.0 ± 0.58	29.67 ± 1.00	0.113
Leucocrit (%)	1.27 ± 0.04	1.24 ± 0.07	1.21 ± 0.07	1.19 ± 0.02	1.23 ± 0.06	0.328

**Notes.**

Values without superscript letters (a, b, c) in the same row are insignificant (*p* < 0.05) determined by one-way ANOVA followed by Duncan’s post hoc multiple range test.

TH tuna hydrolysate FTH fermented tuna hydrolysate Hb haemoglobin Hct haematocrit gdl^−1^ gram per decilitre

Values are the mean of three replicated tanks (*n* = 3) ± standard error.

### Liver histopathology

**Figure 4:**
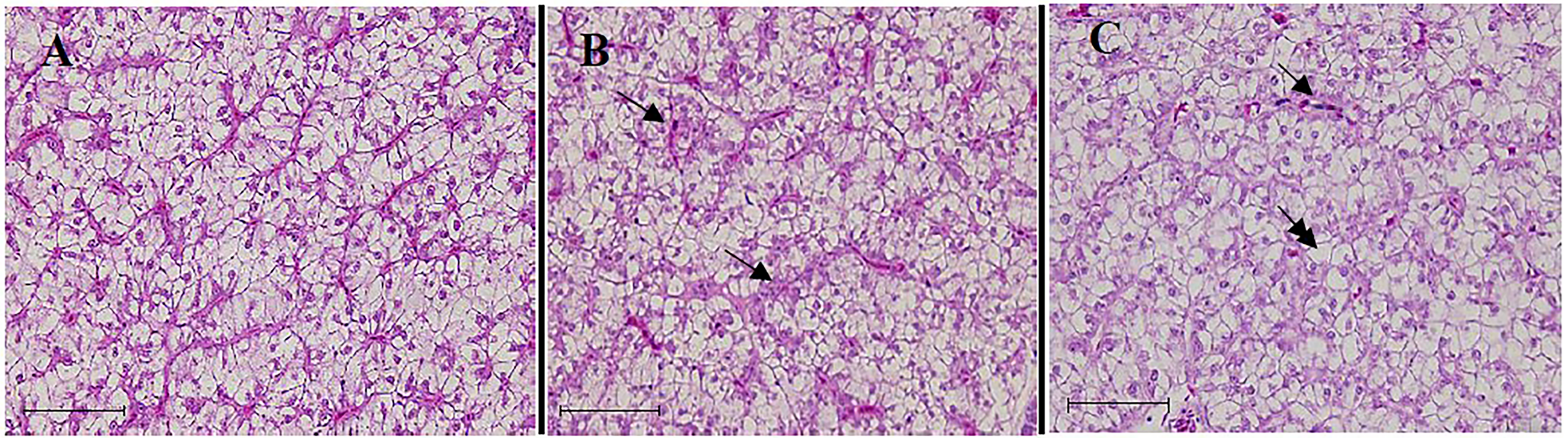
Liver histopathology of juvenile barramundi fed on TH diets without or with fermentation for 56 days. (A) Control group: the hexagonal hepatocyte with predominantly glycogen vacuoles. (B) Fish fed on TH_75_ diet: arrows indicate hepatocytes containing lipid droplet and cellular degeneration. (C) Fish fed on FTH_75_ diet: arrow indicates necrotic foci and double arrow indicates nucleus disappearance in hepatic cells (H & E staining 400× magnification, scale bar = 50 µm).

The control fish exhibited a normal liver condition, which is characterized by hexagonal hepatocytes with a round, central nucleus, and a rare occurrence of cytoplasmic vacuolization or granules (Fig. 4A). The excessive use of TH in the diets resulted in several alterations in the liver tissue of the fish. The alterations, including the occurrence of cytoplasmic vacuolization with lipid accumulation (steatosis), were observed in the fish fed on the TH_75_ diet (Fig. 4B). In the fish fed on the FTH_75_ diet, severe and irreversible damages were observed in the liver, which were characterized by disorganized hepatic cordons, cellular deformation, and nuclear hypertrophy. In further damaged livers, in addition to the other alterations, the absence of nucleolus and the presence of necrotic foci were observed (Fig. 4C).

## Discussion

The dietary inclusion of fish protein hydrolysate (FPH) produced from whole herring (*Clupea harengus*) at moderate levels (18% to 24%) has been reported to confer a positive effect to the growth performance, feed utilization, and digestibility in the Atlantic salmon (*Salmo salar* L.); whereas, the inclusion of the same FPH at higher (>24%) and lower (<12%) levels has been demonstrated to confer negative effects (Hevrøy et al., 2005). In a similar vein, Kim et al. (2014) reported that the replacement of fishmeal in the fish feeds by more than 30%, with tuna by-product ingredients resulted in a decrease in the growth and feed utilization of juvenile olive flounder (*Paralichthys olivaceus*). Similar outcomes were observed by Cahu et al. (1999), where the addition of more than 19% of soluble protein hydrolysate negatively affected the enzymatic activities of trypsin, alkaline phosphatase, and aminopeptidase in the 41-days-old larvae of sea bass (*Dicentrarchus labrax*). The present study supports these findings; herein also, the feeding of juvenile barramundi with diets containing higher levels of TH (50% to 75%) resulted in detrimental effects on the WG and SGR of the fish. These deleterious effects on the growth performance of the fish could be due to an excessive number of short-chain peptides and free amino acids (FAA) present in the hydrolyzed products (Ospina-Salazar et al., 2016), which might have caused saturation in the peptide transport mechanism (Carvalho et al., 2004). Furthermore, the higher amounts of FAA are able to alter the absorption of amino acids, leading to amino acid imbalances in the fish gut (Kolkovski & Tandler, 2000; Rønnestad et al., 2000); this might have been another reason for the poor growth performance of the fish fed on higher levels of protein hydrolysates.

The low-molecular-weight peptides released as a result of protein hydrolysis are often associated with improvement in feed palatability and attractability (Aksnes, Hope & Albrektsen, 2006; Kasumyan & Døving, 2003), which may result in an increased consumption of feed by the fish (Refstie, Olli & Standal, 2004). In contrast, hydrolysis has also been reported to be responsible for creating a bitter taste (of the protein hydrolysate) in the feed, due to the release of certain peptides during the process that contains hydrophobic amino acid residues (FitzGerald & O’Cuinn, 2006). In the present study, a significant reduction in the FI of the fish that were fed on the TH- and FTH-included diets, compared to control, indicated that the differences in the growth performance of the fish belonging to different treatments may be related to decreased palatability or increased bitterness of the diets. An increase in the FCR values was observed with the increasing addition of TH in the diets. In accordance with the present study, a significant increase was observed in the FCR values, in juvenile Japanese sea bass (*Lateolabrax japonicus*) that were fed on diets containing 15%–25% FPH produced from the gut and head of Alaska pollock (*Theragra chalcogramma*) (Liang et al., 2006). Moreover, Hevrøy et al. (2005) demonstrated that the dietary inclusion of FPH procured from whole herring (*Clupea harengus*) at higher levels (>30%), in the diets of Atlantic salmon (*Salmo salar* L.), significantly increased the FCR values for the salmon fish. However, Ospina-Salazar et al. (2016) observed no significant variation in the FCR values, despite feeding the juvenile pike silverside (*Chirostoma estor*) with diets containing up to 45% of fish soluble protein concentrates.

The CF of fish has been reported to reflect the nutritional status of the fish, and act as an indicator of the physiological condition of the fish (Vo et al., 2015). In the present study, the CF values were not influenced by the inclusion levels of TH and FTH in the diets of the juvenile barramundi. Similarly, Ovissipour et al. (2014) were not able to observe any significant difference in the CF of Persian sturgeon (*Acipenser persicus* L.) which was fed on a diet containing tuna-viscera protein hydrolysate at 50% inclusion level. In the present study, the replacement of fishmeal protein by TH and FTH did not influence the HSI and VSI values for the fish. These results were in agreement with the findings reported by Khosravi et al. (2015b), a study which involved red sea bream (*Pagrus major*). However, the results of the present study for VSI values were in contrast with the findings of Xu et al. (2016), who reported a decreased performance in turbot (*Scophthalmus maximus*) which was fed on diets containing higher levels of FPH. The decreased HSI, observed in the present study, with increasing levels of FPH may indicate improper storage of the macro- and micro-nutrients in the fish body, an unhealthy condition of the liver, and clinically an unhealthy sign. Nevertheless, these indices might have been influenced by a variety of factors, including sex, life history, availability of food, and the experimental condition of the fish (Barton, Morgan & Vijayan, 2002).

Several studies have reported that the inclusion of protein hydrolysates in fish diets improves the digestibility of fish (Hevrøy et al., 2005; Zheng et al., 2012); whereas certain other studies have reported that the inclusion of FPH at higher levels may often cause adverse effects on the digestibility of the fish (Ospina-Salazar et al., 2016). In the present study, it was observed that the ADCs for dry matter, protein, and lipid decreased with the increasing levels of TH and FTH inclusion in the diets, which might have occurred due to the availability of excess amounts of free amino acids and free nucleotides, which may, in turn, have disturbed the normal process of digestion and metabolism of the ingested diets, resulting in poor digestibility (Zheng et al., 2013). In a study by Ospina-Salazar et al. (2016), it was observed that the replacement of FM by more than 30% with FPH (CPSP Special-G™; SoproPêche, Boulogne-sur-Mer, France) resulted in a significant reduction of ADCs for dry matter and lipid; however, the ADC for protein remained unaltered by the inclusion levels of FPH in the diets. On the other hand, Bui et al. (2014) and Oliva-Teles, Cerqueira & GoncAlves (1999) reported that the dietary inclusion of FPH in red sea bream (*Pagrus major*) and in turbot (*Scophthalmus maximus*), respectively, caused no influence on the digestibility of the fish.

In the present study, the body composition of the juvenile barramundi remained unaffected by the addition of TH in the diets. Similarly, in the studies conducted by Khosravi et al. (2015b) on sea bream (*Pagrus major*), and Oliva-Teles, Cerqueira & GoncAlves (1999) on turbot (*Scophthalmus maximus*), no differences were observed in the whole-body proximate composition of the fish that were fed on diets containing FPH at different inclusion levels. However, the results of the present study are contradictory to the findings of Ospina-Salazar et al. (2016), who reported a decrease in the lipid composition with an increase in the inclusion level of fish hydrolysate in the diets of juvenile pike silverside. In addition, Kim et al. (2014) reported that a moderate-level (>30%) inclusion of FPH in an FM-based diet significantly elevated the whole-body protein composition in juvenile olive flounder (*Paralichthys olivaceus*). Furthermore, Zeitler, Kirchgessner & Schwarz (1984) reported that body composition was influenced by the species of the fish, the diet formulations, and the feeding method. It is possible that a number of factors, including the source of the hydrolysates, the varying inclusion levels of the hydrolysates, and the fish species, may have influenced the effects of FPH on the whole-body composition of the fish.

Hematological indices are used as important biological indicators for examining the physiological changes and the health condition of fish (Vazquez & Guerrero, 2007). In the present study, no association was observed between the levels of replacement of the FM protein with TH diets and the modulations in the hematological indices, for juvenile barramundi. However, the Hb concentration obtained in the present study was comparable to that reported in a study on Atlantic salmon (*Salmo salar* L.), where the Hb concentration (%) remained unaltered even after feeding the fish with diets containing hydrolysates from whole herring (*Clupea harengus* L.) at 30% inclusion level(Hevrøy et al., 2005). Similarly, the inclusion of hydrolysates from krill, shrimp, and tilapia in the diets did not alter the qualitative descriptions of Hb in red sea bream (*Pagrus major*) (Khosravi et al., 2015b)*.* Furthermore, Ilham & Fotedar (2017) observed no significant variation in the Hb concentration in juvenile barramundi fed on fermented soybean meal supplemented with organic selenium. In the present study, the average hematocrit concentration ranged between 24% and 29.67%, which was below the normal range (30%–45%) suggested by Adams, Brown & Goede (1993). It appears that a number of abnormalities, including stress and disease, in the farmed fish are directly or indirectly related to the physiological changes in the blood system (Ilham, Siddik & Fotedar, 2016). For example, leucocrit content in the blood plays a vital role and serves as an indicator of health status in fish (Wedemeyerr, Goulda & Yasutake, 1983), as malnutrition, as well as lower resistance to pathogens, are associated with lower levels of leucocrit (Ilham, Hapsari & Fotedar, 2018). In the present study, the leucocrit percentage was not influenced by the inclusion of fermented and non-fermented TH in the diets. There is no published information available to compare the differences in the leucocrit levels observed in this study in the juvenile barramundi fed on TH diets.

The enzymatic antioxidant GPx is more potent than any other antioxidant defense enzymes in fish (Ross et al., 2001). GPx plays a crucial role in accelerating the enzymatic defense system against the production of the extreme *reactive oxygen species* (*ROS*), as well as against lipid peroxidation (Kohen & Nyska, 2002). In addition, it protects the cells from the oxidative damage by metabolizing the hydroperoxides (Arthur, McKenziey & Beckett, 2003). The protein hydrolysates produced from fishes such as tuna (*Thunnus obesus*), mackerel (*Scomber australasicus*), and Alaska Pollack (*Theragra chalcogramma*) have demonstrated antioxidant activity (Je, Park & Kim, 2005; Wu, Chen & Shiau, 2003; Yang et al., 2011). In recent years, hydrolyzed proteins from fish have been observed to possess antioxidant properties (Bougatef et al., 2010) and exhibit immunomodulatory effects (Liang et al., 2006). The antioxidant activity has been reported to be influenced by the inclusion levels of the hydrolysates and the composition of free amino acids and peptides in the protein hydrolysates (Wu, Chen & Shiau, 2003). In the present study, increased antioxidant activity was observed in the fish fed on fermented TH compared to the fish fed on non-fermented TH, at 75% replacement level, indicating that fermentation promotes the positive effects of augmenting the antioxidant capacity in the fish. This result is in line with the antioxidant GPx responses to the dietary inclusion of fermented soybean meal (SBM) reported in red sea bream (*Pagrus major*) by Kader et al. (2010). Similarly, a study by Azarm & Lee (2014) reported that fermented soybean meal prompted the enzymatic antioxidant GPx in blackhead sea bream (*Acanthopagrus schlegelii*), through the bioavailability of isoflavones produced by the microbial activity. Moreover, one of our previous studies suggested that an appropriate level of dietary inclusion of fermented lupin meal supports growth and the antioxidant GPx activity in juvenile barramundi (Ilham & Fotedar, 2017). However, in the present study, the reduced GPx activity in the fish fed on TH_50_, FTH_50_, and FTH75 replacement diets compared to control may have been caused as a result of a higher inclusion level of TH rather than due to the fermentation process. The decrease in the antioxidant capacity might have triggered a considerable consequence on the cellular structure of the studied fish. This circumstance might have accelerated the lipid accumulation in the hepatocytes, which could be the possible reason for the reduced growth and feed utilization reported in the juvenile barramundi.

The liver, which is an accessory digestiveorgan, is a good indicator of the nutritional condition of fish (Lozano et al., 2017; Raskovic et al., 2011). The most common changes observed in the fish liver due to nutritional disorders are lipid accumulation, vacuolization in the hepatocytes, changes in the size and shape of the nuclei, changes in liver parenchyma and the cell membranes, disappearance of the nucleolus, and in severe cases, formation of necrotic foci (Raskovic et al., 2011). In the present study, the fish fed on the TH_75_ and FTH_75_ diets revealed certain nutritional deficiency symptoms, including the presence of hepatocytes that contained lipid droplet, and the degeneration of vacuoles, along with necrotic loci and necrosis. It was assumed that an excess amount of FPH in the diets may have been able to boost the tocopheroxyl radicals in the tissues, thereby accelerating lipid peroxidation and tissue damage in the liver of the fish. According to Caballero et al. (1999), lipid deposition in the liver indicates a pathological process that may be considered an indication of hepatic failures in fat metabolism. Moreover, the hepatic alteration, due to excessive caloric ingestion which saturates the physiological competency of the liver, may also lead to lipid accumulation (Spisni et al., 1998). Apart from the lipid accumulation, distorted nucleus, changes in the cell membranes, and mild necrosis in certain regions of the liver are the major symptoms of liver toxicity (Shiogiri et al., 2012).

## Conclusion

Feeding the juvenile barramundi with high levels (50% and 75%) of TH-included diets resulted in declined growth and digestibility in the fish, and abnormal signs of liver histopathology were observed. When the FM protein was replaced with fermented and non-fermented TH at 50% and 75% inclusion levels in the diets, it did not improve the health and the antioxidant capacity of the juvenile fish, as indicated by the biochemical responses of blood and the GPx activity, respectively. However, it is unclear whether fermentation is able to improve the dietary efficacy of TH, which has already been subjected to intensive processing. Therefore, further studies are required to investigate the effects of fermentation and the optimum levels of TH inclusion in the diets for an improved growth performance and blood physiology.

##  Supplemental Information

10.7717/peerj.4870/supp-1 Data S1 Raw data Growth performance, length and weight frequency, body composition, digestibility and blood biochemical composition of juvenile barramundi.

## Additional Information and Declarations

### Data Availability

The following information was supplied regarding data availability: The raw data are provided in a Data S1.
